# The associations between sedentary behaviour and mental health among adolescents: a systematic review

**DOI:** 10.1186/s12966-016-0432-4

**Published:** 2016-10-08

**Authors:** Erin Hoare, Karen Milton, Charlie Foster, Steven Allender

**Affiliations:** 1 Global Obesity Centre (GLOBE), World Health Organization Collaborating Centre for Obesity Prevention, Centre for Population Health Research, Deakin University, 1 Gheringhap Street, Geelong, 3220 Victoria Australia; 2 British Heart Foundation Centre on Population Approaches for Non-Communicable Disease Prevention, Nuffield Department of Population Health, University of Oxford, Oxford, UK

**Keywords:** Adolescents, Mental health, Sedentary behaviour, Screen time

## Abstract

**Background:**

With technological developments and modernised sedentary lifestyles has come an increase in diseases associated with inactivity such as obesity and other non-communicable diseases. Emerging evidence suggests that time spent sedentary may also interact with mental health. This systematic review examined the associations between sedentary behaviour and mental health problems among adolescents.

**Methods:**

This systematic review followed Preferred Reporting Items for Systematic Reviews and Meta-Analyses, and applied a quality assessment tool for quantitative studies to identity best available evidence. Following stringent search strategy of the databases; Cumulative Index to Nursing and Allied Health Literature, Global Health, Health Source: Nursing and Academic Edition, MEDLINE, PsychARTICLES and PsycINFO, we identified 32 articles eligible for review.

**Results:**

All studies reported leisure screen time among adolescents, and two thirds of identified studies examined depressive symptomatology. Other mental health measures were; anxiety symptoms, self-esteem, suicide ideation, loneliness, stress, and psychological distress. Strong consistent evidence was found for the relationship between both depressive symptomatology and psychological distress, and time spent using screens for leisure. Moderate evidence supported the relationship between low self-esteem and screen use. Poorer mental health status was found among adolescents using screen time more than 2–3 h per day, and gender differences exist. Essential information was missing for quality of evidence including heterogeneity in mental health and screen time-based measures, and self-report data collection methods.

**Conclusions:**

The findings are of particular significance given the global public health concern of lifestyle-attributed diseases and the possibility for novel approaches to mental health. Future research should examine the psychological impact of reducing time spent using screens for leisure among adolescents, whilst accounting for possible confounding factors such as physical activity and dietary behaviours. It is critical that the reciprocal relationship between lifestyle behaviours and mental health is represented in both the psychiatric and public health forum.

**Electronic supplementary material:**

The online version of this article (doi:10.1186/s12966-016-0432-4) contains supplementary material, which is available to authorized users.

## Background

Lifestyle behaviours represent a broad public health concern with technological developments and modernised sedentary lifestyles has come an increase in diseases associated with inactivity such as obesity and other non-communicable diseases (NCDs) [1, 2]. In addition to physical health determinants, emerging evidence suggests that time spent sedentary may interact with emotional and mental health outcomes [3]. Sedentary behaviours are any waking behaviours with energy expenditure less than or equal to 1.5 metabolic equivalents while in a sitting or reclining posture [4]. Although not indicative of total daily time spent sedentary, screen-based activities for leisure are considered highly prevalent forms of sedentary behaviour [5]. Adolescence is a period of significant risk for the onset of mental disorders [6]. It is also a time during which independent lifestyle behaviours are developed which can significantly impact on immediate and long-term health [7, 8].

There are several international observational studies examining the relationship between sedentary behaviour and mental health during adolescence. However, there is yet to be a cohesive review of the associations between screen time-based sedentary behaviours and mental health outcomes among adolescents specifically. In contrast there are several systematic reviews of the impact of screen time or sedentary behaviours on a broad range of physical health and educational indicators including; body composition, fitness, academic achievement, sleep problems, and musculoskeletal pain [9, 10].

Two recent systematic reviews synthesised evidence on sedentary behaviour and health (including mental health) among young people [11, 12]. One review [11] focused on objectively measured sedentary behaviour only and while this offered methodological rigour, the literature on associations with mental health is in its infancy and requires broad inclusion criteria such as studies reporting self-reported sedentary behaviour. One other review [12] was the first to comprehensively summarise associations between sedentary behaviour and mental health indicators among young people, however this study had some important limitations. The search strategy included literature on both children and adolescents, however adolescents have previously been identified as a ‘specialist’ group, with unique health risks that require separate targeted review methods [13]. In addition, this review failed to identify some key empirical research (e.g., [14–18]), and did not include experimental studies. Our review aims to extend on this previous work by identifying all relevant literature to date and by specifically examining adolescent groups.

A review published in 2011 examined physical activity and mental health among children and adolescents, and included a brief summary of observational research for sedentary behaviour and mental health [19]. Findings indicated a small, but consistent relationship between screen-time and mental health in young people, with authors predicting that this represents a growing body of research.

More recently, four systematic reviews of the literature have examined mental health and sedentary behaviours across age groups including adolescents, however outcomes of interest were limited to depression [20, 21], anxiety [3], or self-harm and suicide [22].

We feel there is sufficient range of studies that merit synthesis. The objective of this systematic review is to synthesize all available evidence on the associations between different types of sedentary behaviour and mental health among adolescent populations. This overview of the research landscape could offer clearer understanding of the interrelated links between lifestyle behaviours and mental health among the adolescent age group, who are known to experience specific health vulnerabilities.

The research questions answered in this review were:

- 1. What is the current state of the evidence and magnitude of associations between different types of sedentary behaviour and mental health among adolescents?
- 2. What are the limitations of the current evidence base and what recommendations can be made for future research?

## Methods

This study followed Preferred Reporting Items for Systematic Reviews and Meta-Analyses (PRISMA) [23].

### Inclusion/exclusion criteria

Inclusion criteria were designed to identify all previous studies examining the sedentary behaviour and mental disorders and symptomatology among adolescent populations. Adolescence was defined as 10 to 19 years of age [24]. Studies that included data on young people within adult/child populations were analysed and reported where appropriate. Studies of adolescents experiencing general good health were selected due to possible confounding effects of experiencing a chronic physical health condition. Importantly, the negative impact of symptomatology associated with such disorders, despite not necessarily amounting to clinical diagnosis, is known [25]. Therefore the outcome of interest in this review was extended to include both mental disorders and associated symptomatology.

Sedentary behaviour was defined as activities that require little energy expenditure including sitting or lying down (not sleeping) while watching television or playing electronic games, reading, studying, writing or working at a desk or computer (not for schoolwork). This included the use of electronic media for entertainment/leisure such as television, electronic gaming, and computer use, and is henceforth referred to as ‘leisure screen time’. Studies that examined Internet or gaming addiction or other diagnosed sedentary behaviour disorders were not eligible for review. This was due to the complexity of such conditions in the context of mental health, and the focus of this review on habitual sedentary behaviours in typical adolescent populations.

Studies were included in the review if they were; (1) peer-reviewed primary research, (2) reported data on adolescents, (3) included mental health measure/s (diagnostic or symptomatic), (4) included sedentary behaviour or screen time measure/s including TV, gaming, computer/internet use, (5) reported cross-sectional or longitudinal associations, or an intervention study on typically developing adolescents, and, (6) were published from the start of the selected database through to January 2016. Studies were excluded if they were; (1) treatment and management studies, (2) studies examining child or adult populations, (3) studies focused on Internet or gaming addiction, or, (4) studies of specific populations (such as those with a chronic physical condition).

### Search strategy

**Fig. 1:**
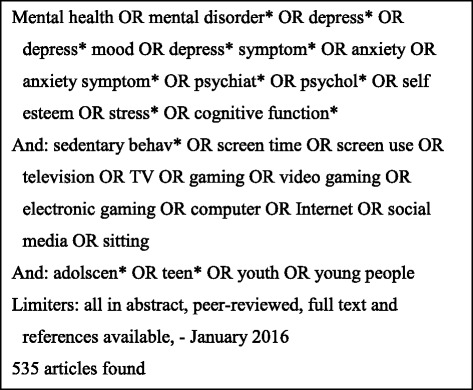
Search terms and strategy

All articles were sourced from databases accessed through EBSCOhost. Databases searched were; Cumulative Index to Nursing and Allied Health Literature (CINAHL), Global Health, Health Source: Nursing and Academic Edition, MEDLINE, PsychARTICLES and PsycINFO. Cochrane Database was searched to identify all possible articles eligible for review. Search terms were selected based on the eligibility criteria and outcomes of interest as previously described. The full search strategy and search terms are reported in Fig. 1.

### Data extraction and synthesis

Two members of the research team [EH, KM] screened the titles and abstracts of all articles identified via the search. Full-text articles deemed eligible for review were sourced and read in full to determine final eligibility. A standardised tool for data extraction was created including study characteristics (author, year, country), participants, study design (longitudinal and intervention studies only), mental health and screen time measures used, and main findings. Findings were categorised into cross-sectional, longitudinal and intervention study designs, and data synthesized by sedentary behaviour measure and mental health outcome examined.

**Fig. 2:**
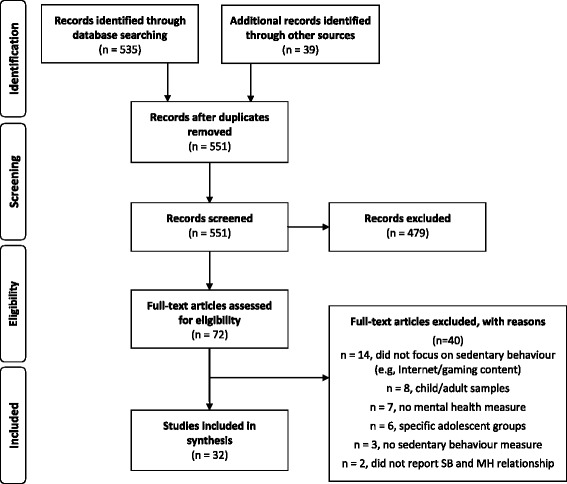
Flow chart for study selection

A total of 535 articles were identified through the search strategy. A further 29 were identified through Cochrane database searching, and 10 through reference list checks of previous systematic reviews. After removing duplicates, all titles and abstracts were screened (*n* = 551) and 72 studies were identified as potentially eligible for the review. Full-text of these 72 articles were sourced and read in full to determine final eligibility. Based on the full-text review, a further 40 articles were excluded; 14 focused specifically on internet content and associated behaviour (such as problem gambling) as opposed to sedentary behaviour specifically, eight examined children or adult populations, seven did not include an appropriate mental health measure, six focused on atypical or specific adolescent groups such as those experiencing a chronic health condition, three did not include an appropriate sedentary behaviour or screen time measure and two compared sedentary behavioural and mental health differences across different groups as opposed to the relationship between the two variables specifically. A total of 32 articles met the eligibility criteria and were subsequently included in this review. A flow chart of study selection process is reported in Fig. 2.

### Quality of evidence

Methodological quality of the included studies was determined by a modified version of a quality assessment tool for quantitative studies [26] as previously used to evaluate evidence for sedentary behaviour and mental health [3]. The tool comprises eight components including selection bias, study design, confounders, blinding, data collection methods, withdrawals and dropouts, intervention integrity, and analyses. Components relevant only to intervention study designs (blinding, intervention integrity) were not applied to cross-sectional and longitudinal studies. Each component was applied to the included studies and graded as weak, moderate or strong, leading to an overall study rating of weak (if two or more components scored weak), moderate (if less than three components were strong with no more than one weak score), or strong (if three or more components were scored strong).

Best evidence synthesis was conducted to draw conclusions for each mental health domain based on the methodological quality of studies. Based on previous systematic reviews examining sedentary behaviour and health outcomes [3, 27, 28], strong evidence was indicated by *consistent* results in 2 or more strong studies. *Consistent* was defined as a stable relationship (in the same direction) that was reported in at least 75 % of studies examining that particular outcome. Moderate evidence was consistent results in one high quality study and at least one weak quality study or consistent results in two or less quality studies. Insufficient evidence was defined as only one available study or inconsistent results in two or more studies.

## Results

### Summary of included studies

Twenty-four of the 32 studies reviewed were cross-sectional in design (Table 1) [29–52], six were longitudinal (Table 2) [14–18, 53] and one was an intervention study design (Table 2) [54]. One study [55] reported both cross-sectional and longitudinal findings. An overview of the type of sedentary behaviour and mental health outcomes that each study examined is reported in Table 3. Details pertaining to study aim, sample characteristics, mental health and sedentary behaviour measures and findings are described in Tables 1 and 2 and will not be repeated in the sections below. Results are arranged by study design, and within sections specific findings are stratified by mental health measure and the sedentary behaviour reported in individual studies.

**Table 1:** Cross-sectional findings

Author, Year, Country of study	Sample characteristics	Mental health measure	Sedentary behaviour measure	Findings	Quality of evidence^a^
Arat, G (2015) [29] United States	Total = 10,563 US school attending adolescents stratified by ethnic background. 13–18 years.	Depression: assessed by one question ‘*during the past 12 months, did you ever feel so sad or hopeless almost every day for two weeks or more in a row that you stopped doing some usual activities?*’ (Yes/No) Suicide ideation: assessed by one question ‘*during the past 12 months, did you ever seriously consider attempting suicide*?’ (Yes/No)	Television: assessed by one question ‘*on an average school day, how many hours do you watch TV?*’	Lower odds of suicide ideation among African adolescents was associated to increased hours spent watching television (OR: 0.82, 95 % CI: 0.72–0.94, *p* < 0.05). No other associations were significant.	Weak
Arbour-Nicitopoulos et al. (2012) [52] Canada	Total: 2,935 Canadian adolescents 13–20 years Mean age: 15.9y (SD:1.4) 49.0 % female	Psychological distress: General Health Questionnaire (Scores ≥3 indicated psychological distress)	Screen time: assessed using one question ‘in the last 7 days, about how many hours a day, on average, did you spend: watching TV/movies, playing video/computer games, on a computer chatting, emailing or surfing the Internet?’ Students categorised into meeting did not met Canadian guidelines of ≤ 2 h per day	Significant associations between exceeding screen time recommendations and psychological distress (OR:1.37, 95 % CI: 1.16–1.62, *p* < 0.001).	Moderate
Asare & Danquah (2015) [31] Ghana	Total: 296 Ghanaian adolescents 13–18 years Mean age: 14.9y (SD:1.4y) 49.3 % female	Depressive symptoms: Child’s Depression Inventory (CDI)	Sedentary behaviour: Adolescents Sedentary Activity Questionnaire to give a daily hourly average spent sedentary. Scores of ≥4 h per day indicated high sedentary behaviour, but also treated as a continuous measure.	Significant positive relationship between sedentary behaviour and depression (*r* = 0.68, *p* < 0.001). Hourly increases in daily average sedentary behaviour was associated to a 0.20 standard deviation increase in depression (b = 0.20, *p* < 0.001).	Strong
Cao et al. (2011) [32] China	Total: 5003 secondary school students from China 11–16 years 47.9 % female ≤2 h screen time per day: 3695 Mean age: 13.1 (SD: 1.0) >2 h per day: 1308 Mean age: 13.3 (SD:1.0)	Depressive symptomatology: Depression Self-rating Scale for Children (Cut-off: 15) Anxiety symptoms: Screen for Child Anxiety Related Emotional Disorders (Cut off: 23)	Screen time: open ended question participants reported how many hours per day, on average they spent on the following sedentary activities outside school hours on a usual weekday, as well as weekend day: TV viewing, computer usage.	High screen time was associated to increased odds for depressive symptoms (OR:1.52, 95 % CI: 1.31–1.76) and anxiety symptoms (OR:1.36, 95 % CI: 1.18–1.57).	Strong
Casiano et al. (2012) [33] Canada	Total: 9137 Canadian youth 12–19 years Mean age not reported (64.9 % aged between 15 to 19y) 48.7 % female	Depressive symptoms: Composite International Diagnostic Interview (CIDI) Short Form (scores were converted to a probability of major depression and cut off of 0.90 was used) Suicide ideation: were considered positive if answered yes to question ‘Have you ever seriously considered committing suicide or taking your own life?’ and ‘Has this happened in the past 12 months?’	Screen time: participants asked the number of hours per week that they had spent using media (including TV/video watching, video game playing, computer/Internet use) in the last three months.	Depression was less frequent in frequent video game users (OR:0.87, 95 % CI: 0.79–0.97, *P* < 0.05).	Moderate
Donchi & Moore (2004) [34] Australia	Total: 336 secondary school and university students 15–21 years, 66.1 % female Secondary students: 110 regional secondary school students 15–18 years Mean age: 16.2y (SD:0.8y) University students: 226, 17–21 years Mean age: 18.6y (SD:1.1y)	Loneliness: UCLA Loneliness Scale, Wittenberg’s 10-item Emotional and Social Loneliness Scale Self-esteem: Form A of the 16-item Texas Social Behaviour Inventory	Internet use: amount of time young people spend on internet on an average day, asking to indicate in minutes time ‘on an average day’ spent on 13 items relating to internet use such as ‘visiting chat rooms’, ‘searching for things of personal interest’, ‘finding articles and references’.	None of the measures for time spent online (categorised into communication, entertainment or information-related activities) were significant predictors of well-being for male or female adolescents.	Moderate
Durkin & Barber (2002) [35] United States	Total: 1304 10^th^ grade secondary school students in 1988 Mean age: 16y (SD not reported) Gender descriptive not reported	Depressed mood: measured with a four-item scale with sample item ‘how often do you feel unhappy, sad, or depressed?’ Self esteem: measured with three items with sample item ‘how often do you feel satisfied with yourself the way you are?’ Responses for both items ranged from 1 (never) to 7 (daily). A mean score was computed. No other details.	Computer game use: measured with two questions about computer use, first asked whether the participant ever used a computer (Yes/No), if response yes then asked how often they used a computer to play games, responses ranged from 1 (never) to 7 (daily). Participants categorised into; none (those who did not use computers at all, as well as those who used computers but never for computer games), low (those who checked 2–5 for frequency of computer use for games) and high (6 or 7 for frequency of computer game play).	Depressed mood varied significantly by computer game use (F(2,1014) = 4.19, *p* < 0.05) with the low use group reporting significantly less depressed mood than the high use group and their peers who did not use computer games. Self-esteem differences by computer game use favoured the low play group over the non players (F(2,1014) = 4.00, *p* < 0.05), neither group differed significantly from the high players.	Weak
Fang et al. (2014) [36] Canada	Total: 152 Canadian Chinese youth 17–24 years, 43.4 % females Mean age not reported 103 aged 17–18 years (67.8 %) 49 aged 19–24 years (32.2 %)	Depressed mood: 5 items from the General Health Questionnaire. Summed scores ranged from 0 to 15 and higher scores reflected higher level of depressed mood. Stress: was measured by one item taken from Perceived Stress Scale, participants were asked to indicate on average their level of stress from 0 (not stressed at all) to 10 (very stressful). Suicide ideation: two items from Ontario Student Drug Use and Health Survey, one example ‘in the past 12 months, did you seriously think about committing suicide or taking your own life?’ Scores ranged from 0–2 (no further description provided) where higher scores indicated a greater level of suicide ideation.	Screen time: total number of hours spent per day on the computer and TV, then two further categories; time spent for school and non school related reasons in the past 7 days	Total amount of time spent in screen time was positively associated with perceived stress (β = 0.32, *p* < 0.01). When youth spent more time for non-school related reasons they were more likely to be depressed (β = 0.32, *p* < 0.05). Those who spent more screen time on school related activities experienced higher stress (β = 0.42, *p* < 0.0001).	Weak
Gross (2004) [37] United States	Total: 261 7^th^ and 10^th^ grade Californian secondary school students 61.7 % female Mean age of 7^th^ graders: 12y (SD:0.4y) Mean age of 10^th^ graders: 15y (SD:0.6y)	Participants completed daily reports before going to sleep at night for 3 days (7^th^ graders) or 4 days (10^th^ graders) Depressive symptoms: Child Depression Inventory Loneliness: UCLA Loneliness scale (‘in school’ was added to enable assessment of loneliness at school specifically) Social anxiety: combining items form two subscales of the Social Anxiety Scale for Adolescents produced a total social anxiety index where higher scores indicated increased levels of social anxiety.	Sedentary behaviours: Participants asked to estimate how much time they spent talking on the phone, watching TV and using the Internet	No association between average daily time online and any mental health measure (all p values > 0.1).	Moderate
Herman et al. (2015) [38] Canada	Total: 7725 Canadian adolescents 12–17 years Mean age not reported 49 % female	Self-rated mental health: one item ‘would you say your mental health in general is excellent, very good, fair or poor?’ Responses were dichotomised to estimate the probability of rating one’s health sub-optimally (good, fair, poor) versus optimally (excellent or very good)	Screen time was assessed via questions; ‘in a typical week in the past 3 months, how much time did you spend on a computer, including playing computer games and using the internet? (not including time spent at work or at school), playing video games, watching TV or videos?’ Responses were summed to give a pre-categorised total weekly screen time from which an upper cut off of 14 h per week was used to denote 2 h/day.	Adolescents exceeding screen time guidelines were 30–50 % more likely to rate their mental health sub-optimally compared to those who met guidelines (males OR:1.34 95 % CI 1.11–1.62, females OR: 1.52 95 % CI: 1.28–1.80).	Strong
Hoare et al. (2014) [39] Australia	Total: 800 Australian secondary students 11–14 years Mean age: 13.1y (SD:0.6y) 55 % female	Depressive symptoms: Short Mood and Feelings Questionnaire Cut off of 10 indicated depressive symptomatology presence	Leisure time screen based behaviour: items relating to TV viewing (including videos and DVDs) and three related to playing video games and using the computer (other than for homework), on a single school day, and Saturday and Sunday, then calculated to provide a daily estimate. Dichotomised into met or exceeded Australian guidelines of 2 h or less per day.	Screen time was associated to presence of depressive symptomatology in males (OR:1.22 SE:0.10, *p* = 0.01) and females (OR:1.12 SE:0.06, *p* = 0.02).	Moderate
Jackson et al. (2010) [40] United States	500 American youth Mean age: 12.2y (SD not reported) 53 % female 11–16 years	Self-esteem: Rosenberg self-esteem scale	Internet, videogame and mobile phone use: how often do they use above 1 = do not use at all, 2 = about once a mnth, 3 = a few times a month, 4 = a few times a week, 5 = everyday for less than 1 h, 6 = everyday for 1–3 h, 7 = everyday for more than three hours.	Adolescents who played videogames more had lower self-esteem than did adolescents who played less frequently (*p* < 0.05)	Moderate
Katon et al. (2010) United States	Total: 2291 American adolescents 13–17 years 1993 without depressive symptoms Mean age: 15.3y (SD:1.3y) 47.8 % female 281 with depressive symptoms Mean age: 15.5 (SD:1.3y) 61.2 % female	Depressive symptoms: Patient Health Questionnaire two item depression scale Cut-off score ≥3 indicated depressive symptoms	Screen time: two questions on hours and minutes spent on a computer and TV watching.	Adolescents with depressive symptoms reported a significantly (p < 0.001) higher amount of average time daily using computer (mean: 1.9 SD:1.7) compared to those without depressive symptoms (mean:1.6 SD:1.4)	Moderate
Kremer et al. (2014) [42] Australia	Total: 8029 Australian young people 10-14 years Mean age: 11.5y (SD:0.8) 52 % female	Depressive symptoms: Short Mood and Feelings Questionnaire Cut off: ≥8 indicated depressive symptoms presence	Screen time: participants reported time spent watching TV and on a computer or playing video games for leisure separately for weekdays and weekend days (‘On school days/weekend days for how many hours do you usually watch TV?’, ‘On school days/weekend days for how many hours do you usually spend on a computer or playing video games such as gamecube, xbox, PS2, PSP, GBA etc.?’; 1 = none; 6 = more than 6 h).	Adolescents who were asymptomatic had a greater proportion who met screen time recommendations compared to those with depressive symptoms (*p* < 0.001). A significant age group x screen time effect was observed (OR:0.77 95 % CI:0.59–0.99, *p* < 0.04) indicating the effect of meeting scree time recommendations on depressive symptoms was moderated by the age of the respondent.	Strong
Maras et al. (2015) [43] Canada	Total: 2482 English speaking grade 7 to 12 57.7 % female	Depressive symptoms: Children’s Depression Inventory Anxiety symptoms: Multidimensional Anxiety scale for Children-10.	Screen time: hours per day of TV, video games, and computer was assessed using the Leisure-Time Sedentary Activities Questionnaire, developed by investigators.	Duration of screen time was associated with severity of depression (β = 0.23, p <0.001) and anxiety (β = 0.07, *p* < 0.01). Video game playing (β = 0.13, p <0.001) and computer use (β = 0.17, *p* < 0.001) but not TV viewing were associated with more severe depressive symptoms. Video game playing (β = 0.11, *p* < 0.001) was associated with severity of anxiety.	Moderate
Mathers et al. (2009) [44] Australia	Total: 925 adolescents Mean age: 16.1y (SD: 1.2y) 13–19 years, 49.6 % female	Psychological distress: Kessler 10	Screen time: duration of electronic media use averaged over 1 to 4 days recalled with the Multimedia Activity recall for Children and Adolescents computerized time-use diary	Adolescents who reported high level of video game use were more likely to report high/very high levels of psychological distress (OR: 1.79 95 % CI: 1.17–2.73, *p* = 0.007) compared to those who did not play games. There was a favourable association between low (OR: 0.58 95 % CI: 0.37–0.91, *p* = 0.02) and high (OR: 0.61 95 % CI: 0.38–0.96, *p* < 0.03) computer use and psychological distress compared to no computer use.	Strong
Messias et al. (2011) [45] United States	Total: 29,941 American adolescents 14–18 years 13,817 adolescents from 2007 Mean age: 16.1y (SD:1.2y) 49.7 % female 16,124 adolescents 2009 Mean age:16.1y (SD:1.2y) 47.9 % female	Sadness: ‘during the past 12 months, did you ever feel so sad or hopeless almost every day for two weeks or more in a row that you stopped doing some usual activities?’ Suicidality: ‘during the past 12 months, did you ever seriously consider attempting suicide?’ ‘during the past 12 months, did you make a plan about how you would attempt suicide?’ ‘During the past 12 months how many times did you actually attempt suicide?’ ‘If you attempted suicide during the past 12 month, did any attempt result in an injury, poisoning, or overdose that had to be treated by a doctor or nurse?’	Screen time: on an average school day, how many hours do you play video or computer games or use a computer for something that is not school work? 7 answers were possible ranging from ‘I do not play video or computer games or use a computer for something that is not school work’ to ‘5 or more hours per day’.	Those reporting moderate game/internet use (1 h or less daily) are significantly less likely to report sadness compared to those reporting no use at all, but no statistics available. Those with video game use between 2–3 h were not different from those reporting no video game use. Those reporting 5 h or more were more likely to experience sadness than those reporting no use. Compared to those reporting no video game use/internet those reporting 5 h or more were more likely to have experienced suicide ideation and made suicidal plans in both 2007 group (ideation OR:1.4 95 % CI: 1.1–1.8; planning OR:1.8 95 % CI: 1.3–2.3), and 2009 group (ideation OR:1.7 95 % CI: 1.3–2.1; planning OR:1.5 95 % CI: 1.1–1.9). All *p* < 0.05.	Moderate
Nihill et al. (2013) [46] Australia	Total: 357 females from 12 secondary schools in New South Wales Mena age: 13.2y (SD:0.5y) 100 % female	Self-esteem: self esteem subscale from Marsh’s Physical Self-Description Questionnaire Example item ‘overall, most things I do turn out well’.	Sedentary behaviour: Adolescent Sedentary Activity Questionnaire included amount of time outside school spent in various sedentary behaviours including watching TV/videos/DVDs, using computers for school and non-school purposes, studying, reading, sitting with friends, using the telephone, listening to or playing music, motorized travel, hobbies and crafts. Screen time: assessed by one item asking how much time was spent watching TV, videos, DVDs and using the computer for non-school purposes.	Significant inverse associations between time spent watching DVDs (B:-0.00304 95 % CI:-0.00542 to -0.00067, *p* < 0.05), playing computer games (B:-0.00171, 95 % CI:-0.00299 to -0.00043, *p* < 0.05), and total screen time (B:-0.00084, 95 % CI : -0.00157 to -0.00012, *p* < 0.05) and self-esteem.	Moderate
Pantic et al. (2012) [47] Serbia	Total: 160 high school students Mean age: 18.02y (SD:0.29) 68.1 % female	Depression: Beck Depression Inventory-II-II Cut off: 0–9 minimal depression, 10–18 mild depression, 19–29 moderate depression and 30–63 severe depression.	Screen time: item asked self-report daily average time spent on watching TV, and time spent on social networking sites.	Significant correlation between depression score and time spent on social networking sites (R = 0.15, *p* < 0.05). No correlation was found between TV viewing and depression.	Moderate
Park (2009) [48] South Korea	Total: 3449 Korean second year middle students Mean age and gender not reported.	Depressive symptoms: based on 6 questions examining symptoms listed in the DSM-4	Internet use: 4 items asking how frequent a respondent used the Internet for chat room or messenger, email, club activities, bulletin board.	Increased risk for depressive symptoms was positively associated with greater use of the internet (OR:1.207 95 % CI:1.043–1.398, *p* < 0.05).	Strong
Robinson et al. (2011) [49] Australia	Total: 1860 Australian adolescents Mean age:14.01y (SD:0.20) 46.9 % female	Mental health: Parent reported Child Behaviour Checklist for Ages 4–18 which provided continuous scores from which quartiles represented level of mental health.	Screen time: participant were asked about their television/video viewing habits and computer use which was categorised into less than two hours per day, 2–4 h per day and more than 4 h per day.	Compared to less than two hours per day, adolescents using screen time 2–4 h per day (β:1.88 95 % CI:0.40–3.36, *p* < 0.05) and those who reported 4 or more hours per day (β:2.80, 95 % CI:1.24–4.36, *p* < 0.005) reported higher mental health scores indicating poorer mental health status.	Strong
Trinh et al. (2015) [50] Canada	Total: 2,660 Ontario, Canadian youth Mean age: 15.8y (SD:1.3y) 52.5 % female	Psychological distress: was measured by the General Health Questionnaire to assess symptoms of anxiety, social dysfunction, and self-esteem. Experiencing at least three of the 12 symptoms indicated elevated psychological distress. Depressive symptoms: were assessed using four items adapted from the Centre for Epidemiologic Studies Depression [76]. Having depressive symptomatology was defined as reporting ‘often’ or ‘always’ on all four symptoms. Higher scores indicated higher depressive symptomatology. Self-esteem: assessed using six items adapted from the Rosenberg Self-Esteem scale, higher scores indicated lower self-esteem.	Screen time: assessed with one question ‘in the last 7 days, about how many hours a day, on average, did you spend watching TV/movies, playing video/computer games, on a computer chatting, emailing or surfing the internet?’ Responses; none, less than one hour, 1–2 h, 3–4 h, 5–6 h a day and 7 or more hours a day. Dichotomised into not meeting (more than 2 h a day) or meeting (two hours or less).	Exceeding screen time recommendations was significantly related to; psychological distress (OR:2.01, 95 % CI:1.40–2.89, *p* < 0.05), low self-esteem (OR:1.32, 95 % CI:1.17–1.49, *p* < 0.05), depressive symptoms (OR:1.92, 95 % CI:1.05–3.54, *p* < 0.05). In males, higher screen time was associated to psychological distress (OR:2.40, 95 % CI:1.63–3.54, *p* < 0.05), low self-esteem (OR:1.31, 95 % CI:1.13–1.53, *p* < 0.05), and depressive symptoms (OR:2.82, 95 % CI = 1.09–7.30, *p* < 0.05). In females, higher screen time was associated with low self-esteem (OR:1.30, 95 % CI:1.10–1.53, *p* < 0.05).	Strong
Ybarra et al. (2005) [56] United States	Total: 1501 American youth 10–17 years Mean age: 14.1y (SD:1.9y) 47.3 % female	Depressive symptomatology: youth were asked about the presence (yes/no) of each of the nine symptoms of depressive disorder based on DSM-IV [77]. Three categories: DSM-IV-like major depressive symptomatology (five or more symptoms), minor depressive symptomatology (3–4 symptoms) and mild or no depressive symptomatology (fewer than three symptoms).	Internet use: asked to estimate the average number of hours per day he or she used the Internet on a typical day of internet use (1–10+ hours). Participants were asked to estimate the average number of days he or she went online in a typical week.	Among females, compared to mild/no depressive symptomatology, using the internet for 3 or more hours per day was related to increased odds of major like depressive symptomatology (OR:3.57 95 % CI:1.70-7.50, *p* < 0.001), and for minor depressive symptomatology (OR:2.19 95 % CI: 1.20–4.01, *p* < 0.01). No significant findings for males.	Moderate
Young et al. (2013) [30] South Korea	Total: 136,589 South Korean secondary school students 13–18 years Mean age not reported. 47.7 % female	Depressive symptoms: response to ‘during the past 12 months, did you ever feel intense sadness or despair that lasted more than two weeks, and that interfered with your life?’ (yes/no) Suicide ideation: response to ‘during the past 12 months, did you ever seriously consider attempting suicide?’ (yes/no)	Internet use: assessed by ‘how many minutes did you spend using the Internet (for non-study purposes) on average each day for the last 30 days?’ Total amount of time for internet use per week was calculated to capture the average daily amount of time for Internet use.	Compared to 0–17 mins average per day of internet use, an increase of internet use up to 124 mins daily average (OR:-0.07 95 % CI:-0.12 to -0.02, *p* < 0.01), reduced the likelihoods of reporting depressive symptoms and suicide ideation, only showing reverse trends 129 mins daily average per day and beyond, with significance at 184–630 mins daily average (OR:0.19 95 % CI:0.14–0.24, *p* < 0.01). Similar patterns observed in suicide ideation. Up to 124 min reduced likelihood of suicide ideation (OR:-0.08, 95 % CI:-0.15 to -0.02, *p* < 0.01), and beyond average 129 min per day increased likelihood of suicide ideation (OR:0.06 95 % CI:0.00-0.12).	Strong

*β* standardised beta coefficient; *B* unstandardized beta coefficient; *CI* confidence interval; *F* analysis of variance; *OR* odds ratio; *R* correlation coefficient; *SD* standard deviation; *SE* standard error; *y* years

^a^ *Quality of evidence* based on assessment tool for quantitative studies [26] including selection bias, study design, confounders, blinding, data collection methods, withdrawals and drop outs, intervention integrity, and analysis. Strong quality of evidence = if three or more components scored strong. Moderate quality of evidence = if less than three components were strong with no more than one weak score. Weak quality of evidence = if two or more components scored weak

**Table 2:** Longitudinal and intervention findings

Author, Year, Country of study	Sample characteristics	Study design	Mental health measure	Sedentary behaviour measure	Findings	Quality of evidence^a^
Longitudinal						
Bickham et al. (2015) [14] United States	Total: 126 young Americans Mean age at baseline:14.0y (SD: not reported) 46.8 % female	1 year follow-up Baseline: 2009	Depressive symptoms: Beck Depression Inventory	Electronic media use: participants were asked to report the typical amount of time on school days and weekends that they used electronic media including TV, video games, computers, mobile phones and music. Calculated a daily average. Also calculated using time use diaries.	Significant positive association between mobile phone use and depression. Longitudinal analyses found that more TV use (b:0.205, *p* < 0.05)and phone use (b:0.177, *p* < 0.10) reported at baseline the higher participants’ depression score was at the 1-year follow-up.	Moderate
Hume et al. (2011) [55] Australia	Total: 155 Australian adolescents Mean age: females 14.4y (SD: 0.64y), males 14.4y (SD: 0.57y) 60 % female	2 year follow-up Baseline: 2004	Depressive symptoms: Centres for Epidemiological Studies Depression Scale for Children Cut point with depressive symptoms: ≥15	Times spent sedentary: accelerometer worn during waking hours for 1 week at the same time of year in 2004 and in 2006 Screen time: self-reported usual TV/video/DVD viewing during a typical week on weekends and weekend days, total was summed to indicate total TV viewing time (min/week)	Females with depressive symptoms in 2004 watched approximately 168 mins/week more TV in 2006 than did those without depressive symptoms. No other relationships were significant. Models accounted for school participants attended.	Strong
Nelson & Gordon-Larsen (2006) [15] United States	Total: 11,957 American adolescents in grades 7–12 Mean age: 15.8y (SD:11.6y) 50 % female	1 year follow-up Baseline: 1994–1995	Self-esteem: Rosenberg Self-esteem Scale	Screen time: adolescents reported watching/playing TV/videos, video or computer games in hours/week.	Adolescents group into clusters and compared to those watching most screen time (sedentary compared to active young people). Active teens were less likely to have low self-esteem.	Moderate
Primack et al. (2009) [16] United States	4142 adolescents in grade 7 through 12 Mean age at follow-up: 21.8y (1.8y) 52.5 % female	7 year follow-up Baseline: 1994	Depressive symptoms: Centres for Epidemiologic Studies-Depression Scale Scores (0–27) summed and used as continuous where higher scores indicated greater severity of symptoms.	Screen time: participants asked to report hours of exposure during the last week to each of 4 types of electronic media: TV, videocassettes, computer games, and radio. Each media type treated as continuous hours per day. Also summed to create overall hours per day.	Those reporting more TV use had significantly greater odds of developing depression (OR:1.08 95 % CI: 1.01-1.16, *p* < 0.05) for each additional hour of daily TV use. Those reporting more total media exposure had greater odds of developing depression (OR:1.05 95 % CI: 1.0004–1.10, *p* < 0.05) for each additional hour of daily use. Females were less likely than males to develop depression given the same total media exposure (OR for interaction term: 0.93 95 % CI: 0.88–0.99, *p* < 0.05)	Moderate
Romer et al. (2013) [17] United States	Total: 719 American youth aged 14–24 years Mean age and gender % not reported.	1 year follow-up Baseline: 2008	Depressive symptoms: one item taken from the Youth Risk Behaviour Survey. Participants asked to indicate the number of times one had experienced ‘≥2 weeks of ‘sadness or hopelessness that interfered with daily activities in the past 12 months’ (once, twice, three times or more)	Screen time: time spent using internet and TV with items that asked for approximate number of hours spent on a typical weekday and weekend using each medium (<1 h, 1–2 h, 3–5 h, 6–8 h, or > 8 h). Converted to a single estimate of weekly use. Video game use assessed with single item asking for time spent on a typical day.	Internet and video game use were associated with increased reports of depression, Controlling for past symptoms and media use, recent depression was associated with greater Internet use, (B:0.119 SE:0.058, *p* < 0.05) and video game playing (B:0.144 SE:0.044, *p* = 0.001).	Moderate
Sund et al. (2011) [53] Norway	Total: 2,464 Norwegian adolescents 12–15 years Mean age at baseline: 13.7y (0.58y) 50.5 % female	1 year follow-up Baseline: 1998	Depressive symptoms: Mood and Feelings Questionnaire total summed score used 0 to 68 where higher scores represent greater severity of symptoms.	Sedentary behaviour: time spent on sedentary activities everyday outside school (e.g., homework, reading, watching TV, games) were assessed in four response categories ranging from ‘less than three hours’ to ‘more than six hours’.	High levels of sedentary activities predicted high depressive symptoms (≥25 score) at follow-up (OR:1.22 95 % CI: 1.02–1.47, *p* < 0.05). A significant sex by sedentary activities interaction effect was found in that sedentary activities was significant only for boys in predicting high scorers (OR:1.53 95 % CI: 1.15–2.03, *p* < 0.05).	Strong
Witt et al. (2011) [18] United States	Total: 592 young Americans Mean age at baseline: 12.2y (SD not reported) 53.6 % female	3-year follow-up. Baseline: 2005	Self-esteem: Rosenberg Self-Esteem scale	Technology frequency of use: participants asked to report their frequency of technology use for a number of items (never, sometimes, often, very often) for video games, general computer use, and communication.	Self-esteem was negatively associated with mean levels of videogame playing and positively associated with computer use.	Moderate
Intervention						
Lubans et al. (2015) [54] Australia	Total: 361 adolescent boys who reported failing to meet international guidelines regarding physical activity or recreational screen time. Mean age: 12.7y (SD:0.5y) Intervention: 181 Control (wait list for ATLAS program): 180	8 month follow-up Baseline: 2012 Intervention design: 20-week school based obesity prevention intervention targeting health behaviours of low-income adolescent boys considered at risk of obesity. Six intervention components, including; parental newsletter focused on limiting recreational screen time and interactive seminars addressing key behavioural messages.	Psychological well-being: measured by 8-item Flourishing Scale. Composite scores of flourishing represent a summary measure of a person’s self-perceived success in areas such as engagement, relationships, self-esteem, meaning, purpose, and optimism.	Screen-time: measured using a modified version of the Adolescent Sedentary Activity Questionnaire asking participants to report total time spent using screens (of any kind) for the purpose of entertainment, on each day of the week.	After adjusting for school and baseline values, the intervention effect on well-being was small but statistically significant (β: 0.10 SE:0.05, *p* = 0.023). The intervention had a positive effect on screen time (β:-0.21 SE:0.06, *p* < 0.001). In the multiple mediator model (including autonomy choice, screen time, muscular fitness, and RT skills competency) changes in screen time was significantly associated to changes in well-being (product of coefficients estimate: 0.038 95 % CI:0.007–0.080, *p* < 0.05.)	Strong

*β* standardised beta coefficient; *B* unstandardized beta coefficient; *CI* confidence interval; *F* analysis of variance; *OR* odds ratio; *SD* standard deviation; *SE* standard error; *y* years

^a^ *Quality of evidence* based on assessment tool for quantitative studies [26] including selection bias, study design, confounders, blinding, data collection methods, withdrawals and drop outs, intervention integrity, and analysis. Strong quality of evidence = if three or more components scored strong. Moderate quality of evidence = if less than three components were strong with no more than one weak score. Weak quality of evidence = if two or more components scored weak

**Table 3:** Summary of outcome measures for mental health and sedentary behaviour

		Mental health					Sedentary behaviour				
	n	Depression	Anxiety	Self-esteem	Suicide ideation	Other mental health	Screen time total	TV	Computer/Internet	Video gaming	Other sedentary behaviour
Cross-sectional											
Arat (2015)	10,563	✔			✔			✔			
Arbour-Nicitopoulos et al. (2012)	2,935					✔	✔				
Asare & Danquah (2015)	296	✔					✔				
Cao et al. (2011)	5003	✔	✔				✔				
Casiano et al. (2012)	9137	✔			✔		✔				
Donchi & Moore (2004)	336			✔		✔			✔		
Durkin & Barber (2002)	1304	✔		✔					✔		
Fang et al. (2014)	152	✔			✔	✔	✔				
Gross (2004)	261	✔	✔			✔	✔				
Herman et al. (2015)	7725					✔	✔				
Hoare et al. (2014)	800	✔					✔				
Hume et al. (2011)	155	✔						✔			✔
Jackson et al. (2010)	500			✔				✔	✔		
Katon et al. (2010)	2291	✔					✔	✔	✔		
Kremer et al. (2014)	8029	✔					✔				
Maras et al. (2015)	2482	✔	✔				✔	✔	✔	✔	
Mathers et al. (2009)	925					✔			✔	✔	
Messias et al. (2011)	29,941				✔	✔	✔	✔	✔		
Nihill et al. (2013)	357			✔			✔	✔	✔		✔
Pantic et al. (2011)	1860	✔					✔				
Park (2009)	3449	✔							✔		
Robinson et al. (2011)	1860					✔	✔				
Trinh et al. (2015)	2,660	✔		✔		✔	✔				
Ybarra et al. (2005)	1501	✔							✔		
Young et al. (2013)	136,589	✔			✔				✔		
Longitudinal											
Bickham et al. (2015)	126	✔					✔	✔	✔	✔	✔
Nelson & Gordon-Larsen (2006)	11,957			✔			✔				
Primack et al. (2009)	4142	✔					✔	✔	✔		✔
Romer et al. (2013)	719	✔						✔	✔	✔	
Sund et al. (2011)	2464	✔					✔				
Witt et al. (2011)	592			✔					✔	✔	✔
Intervention											
Lubans et al. (2015)	361					✔	✔				
Total □✔		20	3	7	5	10	21	10	14	5	5

All studies examined typically developing adolescents aged 10–19 years, except four studies that included young people aged up to 20–24 years [17, 34, 36, 52]. These studies were accepted for review as a large proportion of the samples in these studies met the 10–19 year age requirement. Two thirds (21/32) of studies eligible for review examined depression or depressive symptomatology. Other mental health outcomes were; anxiety, self-esteem, suicide ideation, loneliness, stress, and psychological distress. Results are stratified firstly by mental health measure, then by type of sedentary behaviour measured.

Two thirds of studies (21/32) examined leisure screen time as an overall composite duration (daily/weekly average) or as a dichotomous variable (met or exceeded cut-off). Types of sedentary behaviours examined uniquely were; computer/internet use, television use, and video gaming and all reported sedentary behaviours were for entertainment and leisure. Twelve studies selected for review were conducted in the United States [14–18, 29, 35, 37, 40, 41, 45, 51], eight in Australia [34, 39, 42, 44, 49, 54, 55], six in Canada [33, 36, 38, 43, 50, 52], two in South Korea [30, 48], and one each in: Norway [53], Ghana [31], China [32], and Serbia [47]. There was mostly even representation of gender in most (24/32) reviewed studies with females forming approximately half (43 %–55 %) of samples examined, with exception of four studies that reported samples consisting of two thirds (60 %–68 %) female [34, 37, 47, 55]. Two additional studies examined only female [46] or male populations [54], and two studies did not report gender-related proportions [17, 35].

## Cross-sectional findings

### Depressive symptomatology

Sixteen of the 24 cross-sectional studies included in this review examined associations between depression or depressive symptomatology, and sedentary behaviour [29–33, 35–37, 39, 41–43, 47, 48, 50, 56]. One study [55] included both cross-sectional and longitudinal associations between depressive symptoms and sedentary behaviour and is included here. Three studies controlled for potential confounding effects of self-reported physical activity levels [31, 43, 50].

#### Total daily sedentary behaviour/screen-time use

When sedentary behaviour was examined as average hours spent using screens for leisure per day, four studies identified a significant association between adolescents reporting higher daily average screen time and heightened depressive scores [31, 32, 36, 43]. This finding was shown to be independent of a broad range of potential confounders including; age, gender, relative wealth (socio-economic status, parent’s level of education, geographic location), physical activity levels, weight status, and fruit, vegetable and soft drink consumption.

Studies that treated screen time as a dichotomous measure (whether adolescents met or did not meet an identified daily cut-off) reported higher levels of depressive symptomatology among those exceeding compared to those who met the cut-off (two hours or less per day in all studies) [39, 42, 50]. One study reported this relationship being independent of self-reported physical activity levels [50].

Objectively measured sedentary behaviour (total time spent sedentary measured by accelerometry data) was not associated with depressive symptoms in one study [55]. Another study found a null relationship in a sedentary behaviour variable of combined time talking on the phone, watching TV and using the internet and depressive symptomatology [37].

#### Computer/internet use

One further study identified gender specific findings with females who experienced depressive symptomatology were more likely to use the Internet for three or more hours per day compared to those with mild or no depressive symptomatology [56] and the same relationship was not significant amongst adolescent males in this study.

Low internet use (up to two hours per day) was significantly associated to lower depressive symptoms compared to non-users in one study [30], showing reverse trends once reaching two hours per day, and significantly heightened depressive symptoms at three or more hours of Internet use per day. Another study also reported an increased risk of depressive symptoms with increased Internet use [48] and one other study found a significant correlation between time spent on social networking sites and depressive scores [47].

Durkin and Barber (2002) reported that adolescents who engaged in low computer game use experienced lower depressed mood than both high users of computer games and those who did not use computer games at all [35].

#### TV viewing

One study found that females who experienced depressive symptoms self-reported an average 168 min more TV viewing per week than those without depressive symptoms, and this difference was significant [55]. Arat (2015) found no significant association between TV viewing and depressive symtpomatology among a group of American adolescents [29].

#### Video gaming

Casiano et al. (2012) examined the relationship between media use in the preceding 3 months and found that depression was less common among frequent video game users compared to those who rarely engaged in gaming, before and after controlling for household income and gender [33].

### Anxiety symptomatology

Three cross-sectional studies reported associations between sedentary behaviour and anxiety symptoms [32, 37, 43].

#### Total daily sedentary behaviour/screen-time use

After controlling for potential confounders (gender, relative wealth, weight status, and various diet and activity-related behaviours) two studies [32, 43] identified significantly greater severity of anxiety symptoms among adolescents who reported higher daily average hours of screen time, compared to those reporting lower daily estimates. The third study found no significant relationship [37].

#### Computer/internet use

No studies examined the relationship between anxiety symptoms and computer or internet use.

#### TV viewing

No studies examined the relationship between anxiety symptoms and TV viewing.

#### Video gaming

No studies examined the relationship between anxiety symptoms and video gaming.

### Self-esteem

Five cross-sectional studies examined the relationship between sedentary behaviour and self-esteem [34, 35, 40, 46, 50].

#### Total daily sedentary behaviour/screen-time use

Total time spent using screens for leisure was significantly inversely associated with self-esteem amongst a group of adolescent females, after controlling for age, socio-economic status, BMI, and objectively measured physical activity [46]. An examination of a dichotomous measure of screen time found that adolescents who exceeded an average of two hours per day experienced lower self-esteem than those who averaged two hours or less, and this finding was replicated when males and females were examined separately [50]. This study also controlled for the impact of self-reported physical activity.

#### Computer/internet use

Durkin and Barber (2002) found that low computer users reported higher levels of self-esteem than non-users, however neither group differed significantly in self-esteem from the high computer game users [35]. One study which categorised time spent online by type of activity (communication, entertainment and information-related activities) found no significant associations with self-esteem [34].

#### TV viewing

No studies examined the relationship between self-esteem and TV viewing.

#### Video gaming

Jackson et al. (2010) reported lower self-esteem amongst frequent video game users compared to less frequent gamers [40].

### Suicide ideation

Associations between sedentary behaviour and suicide ideation were examined in five reviewed studies [29, 30, 33, 36, 45].

#### Total daily sedentary behaviour/screen-time use

No significant relationship was detected between screen time and suicide ideation in two studies [33, 36].

#### Computer/internet use

Messias et al. (2011) [45] found that after accounting for age, gender, smoking and self-reported 2-week sadness, adolescents reporting five hours or more of video game/internet use per day were more likely to have experienced suicide ideation compared to adolescents reporting no video game or Internet use. An examination of Internet use among a large (*n* = 136,589) South Korean adolescent sample [30] revealed lower odds of suicide ideation in those using less than two hours per day of Internet use, and those reporting more than two hours experienced an increased likelihood of suicide ideation.

#### TV viewing

Arat et al. (2015) [29] focused on specific ethnicity-related findings, reporting lower odds of suicide ideation among African adolescents who spent longer hours watching television, compared to those who spent less time watching television.

#### Video gaming

No studies examined the relationship between suicide ideation and video gaming.

### Other mental health indicators

Other mental health outcomes examined were; psychological distress [44, 50, 52], loneliness [34, 37], sadness [45], stress [36], self-rated overall mental health status [38], and parent-reported mental health status of their child [49].

#### Total daily sedentary behaviour/screen-time use

Two studies examining Canadian adolescents reported that exceeding 2 h per day average screen time was significantly associated with higher odds of psychological distress compared to two hours or less per day of average screen time [50, 52]. One of these studies [50] demonstrated this relationship to be independent of physical activity levels, and that the association was most pronounced among males. One study found no significant associations between loneliness and total daily average time spent talking on the phone, watching TV and using the Internet [37]. Total amount of time spent using screen time per day was positively associated with perceived stress [36].

Self-rated mental health was negatively associated with total daily screen time in that adolescents reporting more than two hours per day were 30–50 % more likely to rate their mental health sub-optimally (good/fair/poor) [38]. Parent reported mental health status indicated similar trends, with adolescents who spent more than two hours of screen time each day experiencing poorer mental health status compared to those using less than two hours per day [49]. This study controlled for self-reported physical activity levels.

#### Computer/internet use

Mathers et a. (2009) revealed that adolescents who reported low or high computer use experienced lower levels of psychological distress compared to those who reported no computer use. One study examining loneliness reported no significant associations with time spent online (categorised by time spent communicating, entertainment purposes, or information-related activities) [34].

Self-reported sadness was less frequent among adolescents reporting one hour or less of daily game/Internet use compared to those reporting no use at all [45]. In addition, adolescents who reported 5 h or more of video game/Internet use per day were more likely to experience sadness compared to those reporting no use [45].

#### TV viewing

No studies exmained the relationship between TV viewing and other mental health indicators.

#### Video gaming

Among Australian adolescents, after adjusting for gender, age, socio-economic status and BMI z-score, those who reported a high level of video game use were more likely to report high/very high levels of psychological distress compared to those who did not play games [44].

## Longitudinal findings

### Depressive symptomatology

Of the reviewed longitudinal studies, five examined the association between depression or depressive symptoms and sedentary behaviour [14, 16, 17, 53, 55].

#### Total sedentary behaviour/screen time use

Sund et al. (2011) found that time spent on sedentary activities predicted higher scores of depressive symptoms among male adolescents only [53].

#### Computer/internet use

After controlling for past symptoms and media use, Romer et al. (2013) found that recent depression was associated with greater Internet use and video game playing in a cohort of American adolescents [17].

#### TV viewing

Bickham et al. (2015) demonstrated that, examined individually, higher TV use and mobile phone use reported at baseline significantly predicted higher participant depression scores at 1-year follow-up, after controlling for baseline depressive scores [14]. This relationship remained significant following adjustments for gender, ethnicity and parental education.

Similar predictive findings were reported by Primack et al. (2009) in that each additional hour of daily TV use reported at baseline and overall total media exposure, increased the odds of developing depression at 7-year follow-up [16]. A gender interaction was found, suggesting that females are less likely than males to develop depression based on the same total media exposure.

Hume et al. (2011) reported no significant longitudinal relationships between depressive symptoms, objectively measured sedentary behaviour and TV viewing, in a community-based sample of Australian adolescents.

#### Video gaming

No longitudinal studies examined the relationship between video gaming and depressive symptomatology.

### Self-esteem

Two longitudinal studies examined sedentary behaviour and self-esteem [15, 18].

#### Total sedentary behaviour/screen time use

When adolescents were clustered into activity level which included a sedentary group, being categorised in the most sedentary group at baseline predicted lower self-esteem at 1-year follow-up, compared to those categorised as most active [15].

#### Computer/internet use

No longtudinal studies examined the relationship between computer/internet use and self-esteem.

#### Video gaming

In one other study [18] self-esteem at baseline was negatively associated with time spent using video games over a 3-year period, and positively associated with computer use over the same period.

### Intervention findings

One study [54] examined the impact of an intervention on sedentary behaviours and mental health among adolescents.

#### Total sedentary behaviour/screen time use

The intervention program was set in an Australian secondary school and ran for 20-weeks. The aim of the intervention was to target health behaviours of adolescent boys at risk of obesity, with the specific focus on avoiding screen-time. Intervention activities pertaining to sedentary behaviour and mental health specifically were parental newsletter focused on recreational screen time and interactive seminars addressing key behavioural messages. One intervention component was a smart phone application and website for the project. Eight-month follow-up in 2012 found that the intervention had a positive effect on daily screen time, and that reduced screen time was significantly related to improved psychological well-being. Psychological well-being was defined using the Diener et al. (2010) Flourishing Scale [57]. Results were significant after controlling for the school that the participant attended, baseline values, and objectively measured physical activity levels.

### Quality of evidence

Overall methodological quality ratings are reported in Tables 1 and 2 with study characteristics. The outcomes of individual components assessed using the quality assessment tool are provided in Additional file 1. Overall, 12 studies demonstrated strong methodological quality, 17 were rated as moderate and 3 studies demonstrated weak methodology. All reviewed studies demonstrated some methodological weaknesses. There were a large number (23/32) failed to report validity and reliability of measures of both mental health and sedentary behaviour, close to two-thirds (21/32) did not report randomised sampling, and many studies were missing key information such as participation and retention rates. Best evidence synthesis was conducted to determine relative strength of each mental health domain and is summarised here.

Based on consistent findings of seven strong [30–32, 42, 48, 50, 55], seven moderate [14, 16, 17, 39, 41, 43, 51] and two weak [35, 36] studies, the evidence for the positive association between depressive symptoms and sedentary behaviour among adolescents was considered *strong*. Of the three studies that examined anxiety, one strong [32] and one moderate [43] quality study presented a positive association between symptoms and sedentary behaviour. Due to the low number of studies examining this relationship (*n* = 3), and the requirement of consistency to be determined by 75 % of studies, this evidence was considered *insufficient*. Based on one strong [50] and four moderate [15, 18, 40, 46] studies demonstrating an association between low self-esteem and sedentary behaviour, this evidence was considered *moderate*. Due to only three out of five findings (60 %) examining suicide ideation and sedentary behaviour reported significant positive associations, the evidence for this relationship was considered *insufficient*. The evidence for a positive association between psychological distress and sedentary behaviour was indicated by two strong studies [44, 50, 52] and one moderate study, and the evidence for this relationship was rated *strong*. The evidence was considered *insufficient* for the mental health outcomes of loneliness, stress, mental well-being and sadness, due to the low study numbers examining associations with sedentary behaviour.

## Discussion

### Principle findings

While a large body of cross-sectional research was identified for review, there is a lack of longitudinal studies examining sedentary behaviour and mental health, and only one intervention was included in the review. Directionality was not possible to determine due to the large cross-sectional evidence base. Most commonly examined mental health indicators were; depressive symptomatology, anxiety symptoms, self-esteem, suicide ideation, and psychological distress, while a few studies examined loneliness, stress, mental well-being and sadness. Whilst the eligibility criteria allowed for a broad range of sedentary behaviours, the most frequently examined behaviour in this age group is total screen time for leisure/entertainment purpose, and computer/internet use and TV viewing in particular were most frequently examined.

Findings of this review indicate strong evidence for the positive relationship between depressive symptomatology and screen time for leisure among adolescents, based on a mix of high quality cross-sectional and longitudinal studies. There was some evidence to suggest that low levels of screen time for leisure was associated with lower levels of depressed mood, with adverse findings only appearing at more than two to three hours per day of average of screen time [30, 35, 51].

Although few studies reported psychological distress and sedentary behaviour (*n* = 3), the best evidence synthesis suggests that these studies demonstrate strong evidence for a relationship between high levels of screen time (total screen time, video and computer use) and high levels of psychological distress.

Moderate evidence was found for the relationship between self-esteem and sedentary behaviour, indicating lower levels of self-esteem amongst those who reported higher levels of screen time (total screen time, TV viewing, and computer use).

### Potential mechanisms

While the evidence examining lifestyle behaviours and mental health is growing, there is a notable gap exploring and revealing mechanisms underlying such associations [20]. Studies have typically examined associations, and then discussed possible explanations for findings as opposed to being driven by clear theoretical concepts from the outset. One possible explanation may be the complex range of mediators driving lifestyle behaviours, and the difficulties in extrapolating associated impact.

Although evidence of the independent effect of sedentary behaviour on health is emerging, it has been *assumed* that time spent sedentary is linked to physical activity [58]. Physical inactivity has been shown to interact with mental health [19, 59, 60], and exercise has been shown to have a positive effect in mental health treatment studies [61–63]. It is possible that the beneficial pathophysiological, social and general health effects of being active may be omitted when sedentary, which may have a negative impact on mental health. Adolescents who experience poorer mental health may lack motivation to be physically active and may turn to screen based activities requiring little effort as a coping mechanism, and therefore lose such protective effects of physical activity.

Although the relationship between mental health and sedentary behaviour remained significant after controlling for physical activity in six of the reviewed studies here [31, 43, 46, 49, 50, 54], only two used objectively measured physical activity [46, 54], and this relationship requires further investigation. Previous findings have indicated that the relationship between sedentary behaviour and physical activity among children and adolescents is negative, but small [64]. This suggests these behaviours do not directly displace one another, and support the examination of sedentary behaviour as a distinct behaviour. In addition, an adolescent reporting high levels of physical activity might be experiencing poor mental health alongside high levels of sedentary behaviour. The issue of whether physical activity moderates the assoiciation between sedentary behaviour and mental health is beyond the scope of this systematic review, but forms an important avenue for future research.

As with physical activity, there are known complex relationships between sedentary behaviour, overweight/obesity and other associated obesogenic risk behaviours, and this may have a significant negative impact upon adolescent mental health [20]. The relationship between unfavourable body composition [9], weight status [10] and increased levels of sedentary behaviour has been demonstrated among adolescents. Importantly, overweight and obese young people are known to experience particular mental health vulnerabilities including; weight-based teasing and bullying, stigmatisation, poor body image, and self-esteem, and this in turn is expected to impact on potential sedentary behaviour and mental health associations [65]. Weight status/body mass index was included as a covariate in nine of the reviewed studies [30, 32, 38, 39, 43, 44, 46, 50, 53]. It is reccomended that future research includes such weight-based measures as covariates in asessing the relationship between sedentary behaviour and mental health.

There is evidence supporting the role and impact of diet on mental health during adolescence [66]. The relationship between dietary and sedentary behaviours among this age group has also been previously reported [67]. Recently, an updated comprehensive systematic review [68] revealed that among young people, higher levels of sedentary behaviour (TV viewing in particular) is associated with less healthy dietary behaviours such as low fruit and vegetable consumption, and increased consumption of energy dense foods and sugar sweetened beverages. With known impact of nutritional deficiencies on mood [69] and the increased appetite for sweeter and processed foods when experiencing poorer mental health [70], it is possible that diet is implicated in the relationship between leisure screen time and mental health. Diet-related measures were included in four of the reviewed studies [32, 36, 39, 49], and as with weight-based measures, it is recommended that future research include diet as a covairate in assesssing the relationship between sedentary behaviour and mental health.

While outside the scope of this review, the type of activity being undertaken during screen time for leisure may have important implications in the context of adolescent mental health. It has been suggested that increasing time spent sedentary using screens may represent a population increased dependency on social media platforms for connectedness and interaction with peers [71]. Such experiences may hold protective benefits for mental health and an examination beyond time spent sedentary is needed to identify this potential mechanism. Similarly, it has been suggested that the Internet may offer increased access to health information, including mental health support [72, 73], and this relationship requires further investigation. The type of sedentary activity may be appropriate to examine in relation to mental health compared to objectively measured duration spent sedentary. In addition, screen use does not nessesarily equate to sedentary behaviour in metabolic terms and this further supports comprehensive examination of specific experiences in the context of mental health.

### Methodological considerations

In interpreting these findings, it is important to acknowledge that few longitudinal and intervention studies were eligible for review compared to a large body of cross-sectional research, and this limits conclusions on causality. Study design aside, all reviewed studies were missing some essential information required for methodological quality.

Heterogeneity existed in mental health and sedentary behaviour measures, with studies often failing to report validity or reliability estimates for the respective measure of sedentary behaviour. An indicator for screen time has often been derived from participant self-reported response to very few or single items, thus decreasing methodological quality of evidence to date. In addition, there is some debate surrounding the negative health impact of types of sedentary behaviours, and although this is currently being considered and tackled [74], it remains unclear across these pooled studies. This impacts on the specificity of the exposure (based on types of behaviours examined) and consequential evidence synthesis.

Both continuous and categorical measures of mental health have been used thus limiting comparisons across outcomes. Some studies utilised the same tool however employed different cut-off levels and therefore differences exist in the interpretation of mental health outcomes. Although validity and reliability measures were frequently reported for mental health symptomatology, no study examined objective clinical diagnosed mental disorders. Important differences in statistical analysis limited a comparison of findings, as did the varying adjustments for potential confounders.

A final methodological consideration was that although the intervention study design included in this review aimed to reduce leisure screen time, one intervention component included a smart phone application and a website dedicated to the intervention. Although the purpose of this component was to monitor physical activity including recording of fitness challenge results and goal setting for physical activity and screen-time, such initiatives may impact on the sedentary behaviour and mental health relationship under examination.

### Strengths and weaknesses of this review

This review employed stringent systematic review methodology in line with the PRISMA guidelines [23] to ensure all relevant literature to date was identified and evaluated with the best possible scientific rigour. In addition, the conclusions drawn by this review are strengthened by the use of the quality assessment tool [26] and best evidence synthesis consistent with previous literature examining sedentary behaviour and health outcomes [3, 27, 28].

A limitation of this review was that in order to identify all relevant evidence to date, no exclusion criteria were set on size of samples examined in cross-sectional research. Such methods are advised to ensure studies examined represent those large enough and with sufficient power to detect significant change. This limitation was accepted as necessary due to sedentary behaviour and mental health research in this age group being in its infancy. In addition, our review was not able to infer on the direction and magnitude of the associations between sedentary behaviour and mental health problems among adolescents, due to a lack of longitudinal research in this area.

Although the rigorous search strategy aimed to identify all relevant literature, mental health outcomes emerged from the search strategy a posteriori. Various symptoms are associated with mental disorders and it is plausible that some literature may have been overlooked. In addition, literature demonstrating the relationship between sedentary behaviour and physiological responses that are also associated with stress and mood responses (such as hypothalamic-pituitary-adrenal axis activity [75]) may have also been overlooked. Due to heterogeneity of the outcome measures a meta-analysis was not possible in this review.

## Conclusion

Although limited by varying quality of the methodological design of the included studies, this systematic review suggests there is emerging evidence to link increased time spent using screens for leisure, with poorer mental health experiences among adolescents. Specifically, strong consistent evidence suggests higher levels of screen time for leisure or entertainment is associated with heightened depressive symptomatology and psychological distress among this age group. Methodological considerations include heterogeneity in sedentary behaviour and mental health measures, and few longitudinal and intervention study designs. Future research should examine the psychological impact of reducing time spent using screens for leisure among adolescents, whilst accounting for possible confounding factors such as physical activity and dietary behaviours. The challenge remains to tease out the complex interaction between types of sedentary behaviours, i.e., what is being performed while sedentary (e.g., Video game v reading), their physiological and cognitive impacts, and pathways to mental health. The opportunities for sedentary behaviours have increased rapidly and older studies may have been confined to few sedentary behaviours, e.g., TV viewing, however the ability of adolescents to utilize these greater opportunities at younger ages may contribute more exposure to impacts on mental health. Further analysis of time use data may offer more depth to any secular shifts of type and exposure.

The current findings are of particular significance with increasing burden of poor mental health among adolescents, and growing understanding of the negative health impacts of sedentary lifestyles. It is critical that the reciprocal relationship between mental health and lifestyle behaviour is represented in the public health discussion.

## Additional file

Additional file 1: Methodological quality assessment checklist (XLSX 15 kb)

## References

[1] Dale H, Brassington L, King K (2014). The impact of healthy lifestyle interventions on mental health and wellbeing: a systematic review. Ment Health Rev J.

[2] Choi BC, Hunter DJ, Tsou W, Sainsbury P (2005). Diseases of comfort: primary cause of death in the 22nd century. J Epidemiol Community Health.

[3] Teychenne M, Costigan SA, Parker K (2015). The association between sedentary behaviour and risk of anxiety: a systematic review. BMC Public Health.

[4] Cart L (2012). Letter to the editor: standardized use of the terms “sedentary” and “sedentary behaviours”. Appl Physiol Nutr Metab.

[5] Australian Bureau of Statistics: Australian health survey: physical activity. 2011–12. In*.* Canberra; 2015.

[6] Kessler RC, Amminger GP, Aguilar‐Gaxiola S, Alonso J, Lee S, Ustun TB (2007). Age of onset of mental disorders: a review of recent literature. Curr Opin Psychiatry.

[7] Biddle SJ, Gorely T, Stensel DJ (2004). Health-enhancing physical activity and sedentary behaviour in children and adolescents. J Sports Sci.

[8] Eaton DK, Kann L, Kinchen S, Shanklin S, Flint KH, Hawkins J, Harris WA, Lowry R, McManus T, Chyen D (2011). Youth risk behavior surveillance-United States. Morb Mortal Wkly Rep Surveill Summ (Washington, DC: 2002) 2012.

[9] Tremblay MS, LeBlanc AG, Kho ME, Saunders TJ, Larouche R, Colley RC, Goldfield G, Gorber SC (2011). Systematic review of sedentary behaviour and health indicators in school-aged children and youth. Int J Behav Nutr Phys Act.

[10] Costigan SA, Barnett L, Plotnikoff RC, Lubans DR (2013). The health indicators associated with screen-based sedentary behavior among adolescent girls: a systematic review. J Adolesc Health.

[11] Cliff DP, Hesketh K, Vella SA, Hinkley T, Tsiros MD, Ridgers ND, Carver A, Veitch J, Parrish AM, Hardy LL (2016). Objectively measured sedentary behaviour and health and development in children and adolescents: systematic review and meta‐analysis. Obesity Reviews.

[12] Suchert V, Hanewinkel R, Isensee B (2015). Sedentary behavior and indicators of mental health in school-aged children and adolescents: A systematic review. Prev Med.

[13] Steel Z, Marnane C, Iranpour C, Chey T, Jackson JW, Patel V, Silove D (2014). The global prevalence of common mental disorders: a systematic review and meta-analysis 1980–2013. Int J Epidemiol.

[14] Bickham DS, Hswen Y, Rich M (2015). Media use and depression: exposure, household rules, and symptoms among young adolescents in the USA. Int J Public Health.

[15] Nelson MC, Gordon-Larsen P (2006). Physical activity and sedentary behavior patterns are associated with selected adolescent health risk behaviors. Pediatrics.

[16] Primack BA, Swanier B, Georgiopoulos AM, Land SR, Fine MJ (2009). Association between media use in adolescence and depression in young adulthood: a longitudinal study. Arch Gen Psychiatry.

[17] Romer D, Bagdasarov Z, More E (2013). Older versus newer media and the well-being of United States youth: results from a national longitudinal panel. J Adolesc Health.

[18] Witt EA, Massman AJ, Jackson LA (2011). Trends in youth’s videogame playing, overall computer use, and communication technology use: The impact of self-esteem and the Big Five personality factors. Comput Hum Behav.

[19] Biddle SJ, Asare M (2011). Physical activity and mental health in children and adolescents: a review of reviews. Br J Sports Med.

[20] Hoare E, Skouteris H, Fuller-Tyszkiewicz M, Millar L, Allender S: Associations between obesogenic risk factors and depression among adolescents: a systematic review. Obesity Reviews 2013:n/a-n/a. 10.1111/obr.12069 23980942

[21] Liu M, Wu L, Yao S (2015). Dose–response association of screen time-based sedentary behaviour in children and adolescents and depression: a meta-analysis of observational studies. Br J Sports Med.

[22] Daine K, Hawton K, Singaravelu V, Stewart A, Simkin S, Montgomery P (2013). The power of the web: a systematic review of studies of the influence of the internet on self-harm and suicide in young people.

[23] Moher D, Liberati A, Tetzlaff J, Altman DG (2009). Preferred reporting items for systematic reviews and meta-analyses: the PRISMA statement. Ann Intern Med.

[24] World Health Organization. Young people’s health-a challenge for society. Report of a WHO Study Group on young people and “Health for All by the Year 2000”. In: Technical Report Series. Geneva: World Health Organization; 1986. p. 117. 3085358

[25] Ayuso-Mateos JL, Nuevo R, Verdes E, Naidoo N, Chatterji S (2010). From depressive symptoms to depressive disorders: the relevance of thresholds. Br J Psychiatry.

[26] National Collaborating Centre for Methods and Tools. Quality Assessment Tool for Quantitative Studies. Hamilton: McMaster University Hamilton; 2008.

[27] Proper KI, Singh AS, Van Mechelen W, Chinapaw MJ (2011). Sedentary behaviors and health outcomes among adults: a systematic review of prospective studies. Am J Prev Med.

[28] Chinapaw M, Proper K, Brug J, Van Mechelen W, Singh A (2011). Relationship between young peoples’ sedentary behaviour and biomedical health indicators: a systematic review of prospective studies. Obes Rev.

[29] Arat G (2015). Emerging protective and risk factors of mental health in Asian American students: findings from the 2013 Youth Risk Behavior Survey. Vulnerable Child Youth Stud.

[30] Do YK, Shin E, Bautista MA, Foo K (2013). The associations between self-reported sleep duration and adolescent health outcomes: What is the role of time spent on Internet use?. Sleep Med.

[31] Asare M, Danquah SA (2015). The relationship between physical activity, sedentary behaviour and mental health in Ghanaian adolescents. J Child Adolesc Psychiatr Ment Health Nurs.

[32] Cao H, Qian Q, Weng T, Yuan C, Sun Y, Wang H, Tao F (2011). Screen time, physical activity and mental health among urban adolescents in China. Prev Med.

[33] Hygiea Casiano M, DJK MA, Katz LY, Chartier MJ (2012). Media use and health outcomes in adolescents: findings from a nationally representative survey. J Can Acad Child Adolesc Psychiatry.

[34] Donchi L, Moore S (2004). It’s a boy thing: The role of the Internet in young people’s psychological wellbeing. Behav Change.

[35] Durkin K, Barber B (2002). Not so doomed: Computer game play and positive adolescent development. J Appl Dev Psychol.

[36] Fang L, Zhang VF, Poon HLM, Fung WLA, Katakia D (2014). Lifestyle Practices, Psychological Well-Being, and Substance Use among Chinese-Canadian Youth. J Ethnic Cult Divers Soc Work.

[37] Gross EF (2004). Adolescent Internet use: What we expect, what teens report. J Appl Dev Psychol.

[38] Herman KM, Hopman WM, Sabiston CM (2015). Physical activity, screen time and self-rated health and mental health in Canadian adolescents. Prev Med.

[39] Hoare E, Millar L, Fuller-Tyszkiewicz M, Skouteris H, Nichols M, Jacka F, Swinburn B, Chikwendu C, Allender S (2014). Associations between obesogenic risk and depressive symptomatology in Australian adolescents: a cross-sectional study. J Epidemiol Community Health.

[40] Jackson LA, von Eye A, Fitzgerald HE, Zhao Y, Witt EA (2010). Self-concept, self-esteem, gender, race and information technology use. Comput Hum Behav.

[41] Katon W, Richardson L, Russo J, McCarty CA, Rockhill C, McCauley E, Richards J, Grossman DC (2010). Depressive symptoms in adolescence: the association with multiple health risk behaviors. Gen Hosp Psychiatry.

[42] Kremer P, Elshaug C, Leslie E, Toumbourou JW, Patton GC, Williams J (2014). Physical activity, leisure-time screen use and depression among children and young adolescents. J Sci Med Sport.

[43] Maras D, Flament MF, Murray M, Buchholz A, Henderson KA, Obeid N, Goldfield GS (2015). Screen time is associated with depression and anxiety in Canadian youth. Prev Med.

[44] Mathers M, Canterford L, Olds T, Hesketh K, Ridley K, Wake M (2009). Electronic media use and adolescent health and well-being: cross-sectional community study. Acad Pediatr.

[45] Messias E, Castro J, Saini A, Usman M, Peeples D (2011). Sadness, suicide, and their association with video game and internet overuse among teens: results from the youth risk behavior survey 2007 and 2009. Suicide Life Threat Behav.

[46] Nihill GFJ, Lubans DR, Plotnikoff RC (2013). Associations between sedentary behavior and self-esteem in adolescent girls from schools in low-income communities. Ment Health and Phys Act.

[47] Pantic I, Damjanovic A, Todorovic J, Topalovic D, Bojovic-Jovic D, Ristic S, Pantic S (2012). Association between online social networking and depression in high school students: Behavioral physiology viewpoint. Psychiatr Danub.

[48] Park S (2009). The association between Internet use and depressive symptoms among South Korean adolescents. J Spec Pediatr Nurs.

[49] Robinson M, Kendall GE, Jacoby P, Hands B, Beilin LJ, Silburn SR, Zubrick SR, Oddy WH (2011). Lifestyle and demographic correlates of poor mental health in early adolescence. J Paediatr Child Health.

[50] Trinh L, Wong B, Faulkner GE (2015). The Independent and Interactive Associations of Screen Time and Physical Activity on Mental Health, School Connectedness and Academic Achievement among a Population-Based Sample of Youth. J Can Acad Child Adolesc Psychiatry.

[51] Ybarra ML, Alexander C, Mitchell KJ (2005). Depressive symptomatology, youth Internet use, and online interactions: A national survey. J Adolesc Health.

[52] Arbour-Nicitopoulos KP, Faulkner GE, Irving HM (2012). Multiple health-risk behaviour and psychological distress in adolescence. J Can Acad Child Adolesc Psychiatry / Journal de l’Académie canadienne de psychiatrie de l’enfant et de l’adolescent.

[53] Sund AM, Larsson B, Wichstrom L (2011). Role of physical and sedentary activities in the development of depressive symptoms in early adolescence. Soc Psychiatry Psychiatr Epidemiol.

[54] Lubans DR, Smith JJ, Morgan PJ, Beauchamp MR, Miller A, Lonsdale C, Parker P, Dally K (2015). Mediators of psychological well-being in adolescent boys. J Adolesc Health.

[55] Hume C, Timperio A, Veitch J, Salmon J, Crawford D, Ball K (2011). Physical activity, sedentary behavior, and depressive symptoms among adolescents. J Adolesc Health.

[56] Ybarra ML (2004). Linkages between depressive symptomatology and Internet harassment among young regular Internet users. Cyberpsychol Behav.

[57] Diener E, Wirtz D, Tov W, Kim-Prieto C, Choi D-w, Oishi S, Biswas-Diener R (2010). New well-being measures: Short scales to assess flourishing and positive and negative feelings. Soc Indic Res.

[58] Katzmarzyk PT (2010). Physical activity, sedentary behavior, and health: paradigm paralysis or paradigm shift?. Diabetes.

[59] Lucas M, Mekary R, Pan A, Mirzaei F, O’Reilly ÉJ, Willett WC, Koenen K, Okereke OI, Ascherio A (2011). Relation between clinical depression risk and physical activity and time spent watching television in older women: a 10-year prospective follow-up study. Am J Epidemiol.

[60] Harvey SB, Hotopf M, Øverland S, Mykletun A (2010). Physical activity and common mental disorders. Br J Psychiatry.

[61] Schuch FB, Deslandes AC, Stubbs B, Gosmann NP, da Silva CTB, de Almeida Fleck MP (2016). Neurobiological effects of exercise on major depressive disorder: A systematic review. Neurosci Biobehav Rev.

[62] Schuch FB, Vancampfort D, Richards J, Rosenbaum S, Ward PB, Stubbs B (2016). Exercise as a treatment for depression: a meta-analysis adjusting for publication bias. J Psychiatr Res.

[63] Stubbs B, Vancampfort D, Rosenbaum S, Ward PB, Richards J, Ussher M, Schuch FB (2015). Challenges Establishing the Efficacy of Exercise as an Antidepressant Treatment: A Systematic Review and Meta-Analysis of Control Group Responses in Exercise Randomised Controlled Trials. Sports Med.

[64] Pearson N, Braithwaite RE, Biddle SJ, Sluijs EMF, Atkin AJ (2014). Associations between sedentary behaviour and physical activity in children and adolescents: a meta‐analysis. Obes Rev.

[65] Russell-Mayhew S, McVey G, Bardick A, Ireland A (2012). Mental health, wellness, and childhood overweight/obesity. J Obes.

[66] O’Neil A, Quirk SE, Housden S, Brennan SL, Williams LJ, Pasco JA, Berk M, Jacka FN (2014). Relationship between diet and mental health in children and adolescents: a systematic review. Am J Public Health.

[67] Pearson N, Biddle SJ (2011). Sedentary behavior and dietary intake in children, adolescents, and adults: a systematic review. Am J Prev Med.

[68] Hobbs M, Pearson N, Foster PJ, Biddle SJ (2014). Sedentary behaviour and diet across the lifespan: an updated systematic review. Br J Sports Med.

[69] Jacka F, Berk M (2007). Food for thought. Acta Neuropsychiatrica.

[70] Van Strien T, Cebolla A, Etchemendy E, Gutiérrez-Maldonado J, Ferrer-García M, Botella C, Baños R (2013). Emotional eating and food intake after sadness and joy. Appetite.

[71] Shapiro LAS, Margolin G (2014). Growing up wired: Social networking sites and adolescent psychosocial development. Clin Child Fam Psychol Rev.

[72] Horgan A, Sweeney J (2010). Young students’ use of the Internet for mental health information and support. J Psychiatr Ment Health Nurs.

[73] Thackeray R, Crookston BT, West JH (2013). Correlates of health-related social media use among adults. J Med Internet Res.

[74] Chastin SFM, Schwarz U, Skelton DA (2013). Development of a consensus taxonomy of sedentary behaviors (SIT): report of Delphi round 1. PLoS One.

[75] Martikainen S, Pesonen A-K, Lahti J, Heinonen K, Pyhälä R, Tammelin T, Kajantie E, Strandberg TE, Reynolds RM, Räikkönen K (2014). Physical activity and hypothalamic-pituitary-adrenocortical axis function in adolescents. Psychoneuroendocrinology.

[76] Radloff LS (1977). THE CENTER FOR EPIDEMIOLOGIC STUDIES DEPRESSION SCALE A SELF REPORT DEPRESSION SCALE FOR RESEARCH IN THE GENERAL POPULATION. Appl Psychol Measur.

[77] Association AP (2000). Diagnostic and statistical manual-text revision (DSM-IV-TRim, 2000): American Psychiatric Association.

